# Disseminated Mucormycosis with Extensive Cardiac Involvement

**DOI:** 10.7759/cureus.4760

**Published:** 2019-05-27

**Authors:** Mohanad Soliman, Cameron Harding, Hanine El Haddad, Akila Mansour, Michael Anstead

**Affiliations:** 1 Internal Medicine, University of Kentucky College of Medicine, Lexington, USA; 2 Infectious Disease, University of Kentucky College of Medicine, Lexington, USA; 3 Pathology, University of Kentucky College of Medicine, Lexington, USA

**Keywords:** disseminated infection, invasive mucormycosis, cardiac imaging, opportunistic infection, amphotericin b

## Abstract

Mucormycosis is an opportunistic fungal infection. Cardiac involvement is a rare, yet fatal, complication that can occur in disseminated disease. A strong index of suspicion is necessary for prompt treatment, especially in high-risk patients. We present a 62-year-old male patient with a history of diabetes and acute myeloid leukemia; he had pulmonary mucormycosis that was complicated by cardiac involvement as part of disseminated mucormycosis syndrome.

## Introduction

The first case of human infection by mucormycosis was described in 1876 by Fuerbringer [1]. It is a ubiquitous organism that exists in soil and is most commonly transmitted by the inhalation or ingestion of spores. The hallmark of the disease is angioinvasion, leading to thrombosis, infarction, or fatal bleeding of the involved tissue due to the special affinity of mucormycosis with endothelial cells [2]. Disseminated mucormycosis is the least common and the worst prognosis type, and it mostly starts with lesions in the lungs and then disseminates to other sites. Cardiac dissemination of pulmonary mucormycosis, like in our case, is unusual and often diagnosed postmortem; an antemortem diagnosis is very rare and only four such cases have been reported till 2017 [3-6]. We present the case of an immunocompromised patient with cardiac involvement as part of disseminated mucormycosis syndrome.

## Case presentation

The patient is a 62-year-old Caucasian male with insulin-dependent diabetes mellitus (DM) who was diagnosed with high-risk acute myeloid leukemia (AML) three months prior to presentation to the emergency department with a fever of 38.7 degrees Celsius. He received induction with "7+3" (idarubicin and cytarabine) and midostaurin, and he achieved complete remission. His prophylactic antimicrobial regimen consisted of levofloxacin, acyclovir, and posaconazole with appropriate serum levels. His induction course was complicated by febrile neutropenia. Workup was significant for ground-glass opacities in the bilateral upper lobes and small bilateral pleural effusions on chest computed tomography (CT). He underwent bronchoscopy with bronchoalveolar lavage, with no microbiological culture growth. Blood and sputum cultures, respiratory virus polymerase chain reaction (PCR), and Pneumococcal and Legionella urinary antigen were negative. He was treated with antibiotics for possible pneumonia with minimal improvement in symptoms. Repeat chest CT for non-resolving cough showed interval development of pericardial effusion. Pericardiocentesis was performed, with negative cultures and cytology. The pericardial effusion was thought to be due to midostaurin use during the chemotherapy induction phase. He was discharged to a physical rehabilitation facility on oral antibiotics.

**Figure 1:**
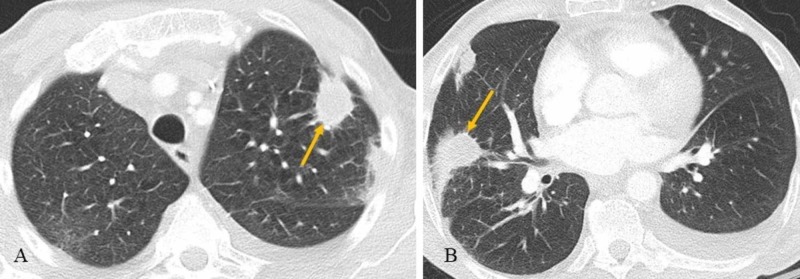
Axial (A and B) chest CT Multifocal bilateral airspace disease with areas of interval cavitation (arrows), consistent with evolving and partially treated sequela of multifocal infection. There are also small bilateral pleural effusions, greater on the left.

One week later, the patient presented with bilateral pleuritic chest pain, dyspnea, and productive cough with brown sputum. His temperature was 38.27 degrees Celsius, blood pressure 98/59 mm/Hg, heart rate 72 beats per minute, with oxygen saturation of 88% on a 4 L nasal cannula. He appeared calm and in no acute distress. There were diminished breath sounds bilaterally on pulmonary auscultation with diffuse crackles. Heart sounds were normal; no murmur, gallop, or rub was appreciated. There was no edema of the lower extremities. The abdomen was soft, non-distended, and non-tender. His white blood cell (WBC) was 13.7 k/uL and hemoglobin was 6.2 g/dL. Fungal serology, beta glucan, galactomannan, Histoplasma urinary antigen, and cryptococcal serum antigen were negative. CT chest showed the progression of a previously identified multifocal bilateral airspace disease with areas of interval cavitation and a loculated fluid collection at the level of the anterior-inferior right atrium (Figure 1).

**Figure 2:**
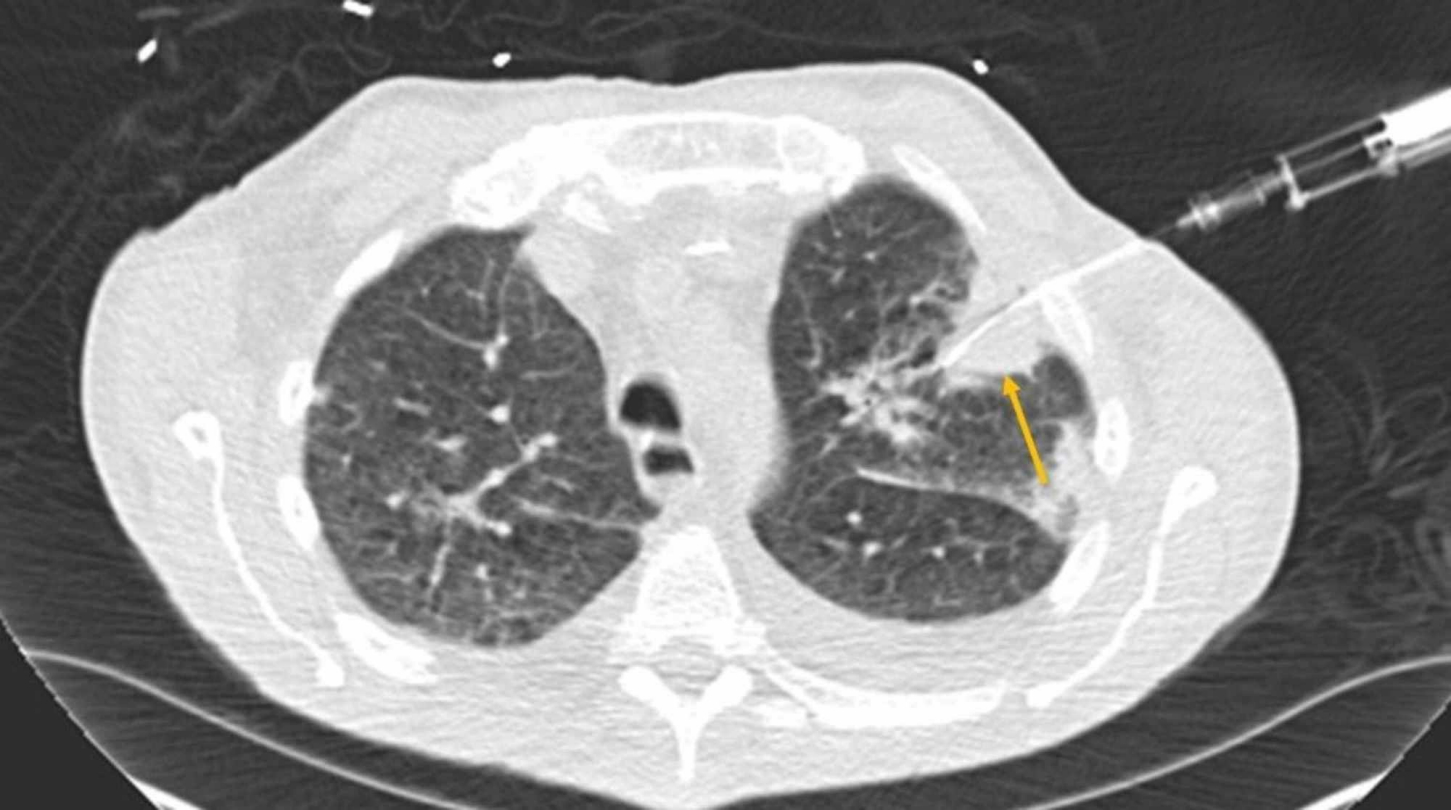
Axial chest CT CT-guided biopsy of a left upper lobe cavitary lesion (the yellow arrow) CT: computed tomography

**Figure 3:**
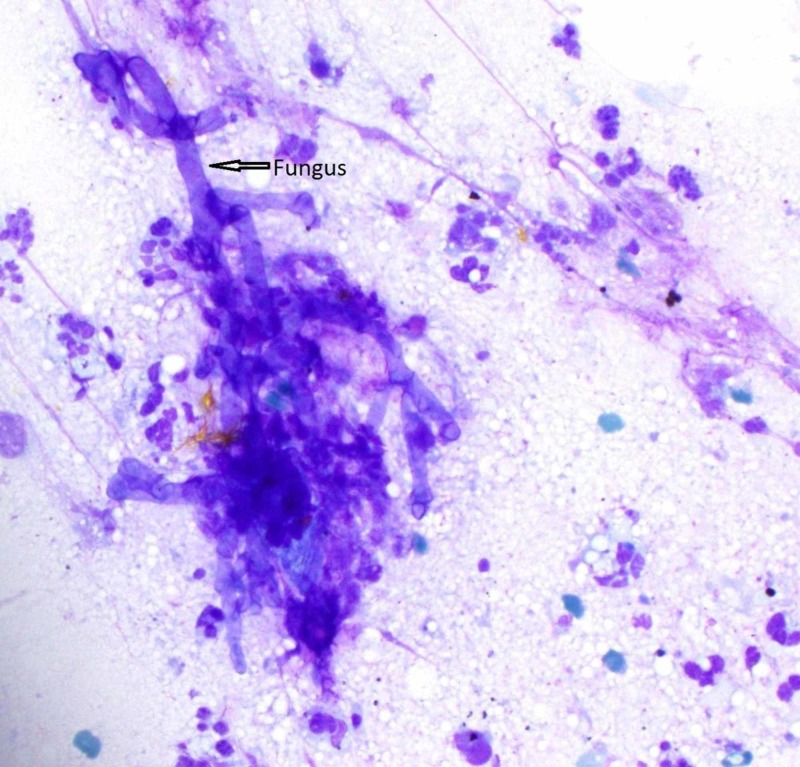
Left lung nodule touch prep 40x Broad, aseptate, ribbon-like hollow hyphae with right angle branching, compatible with zygomycete species

**Figure 4:**
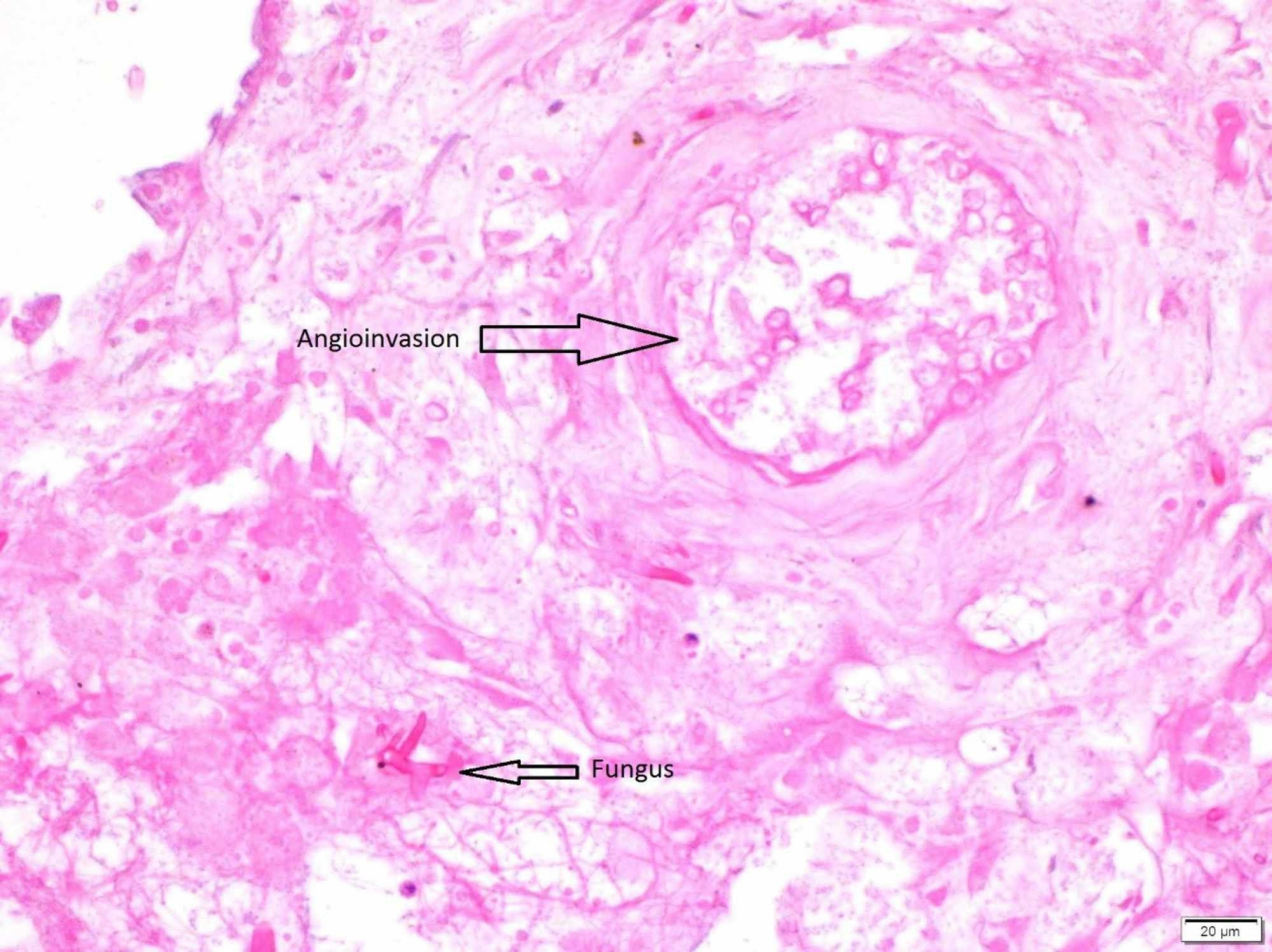
Left lung nodule hematoxylin and eosin stain (H&E) stain Angioinvasion by broad, aseptate, ribbon-like hollow hyphae with right angle branching, compatible with zygomycete species

**Figure 5:**
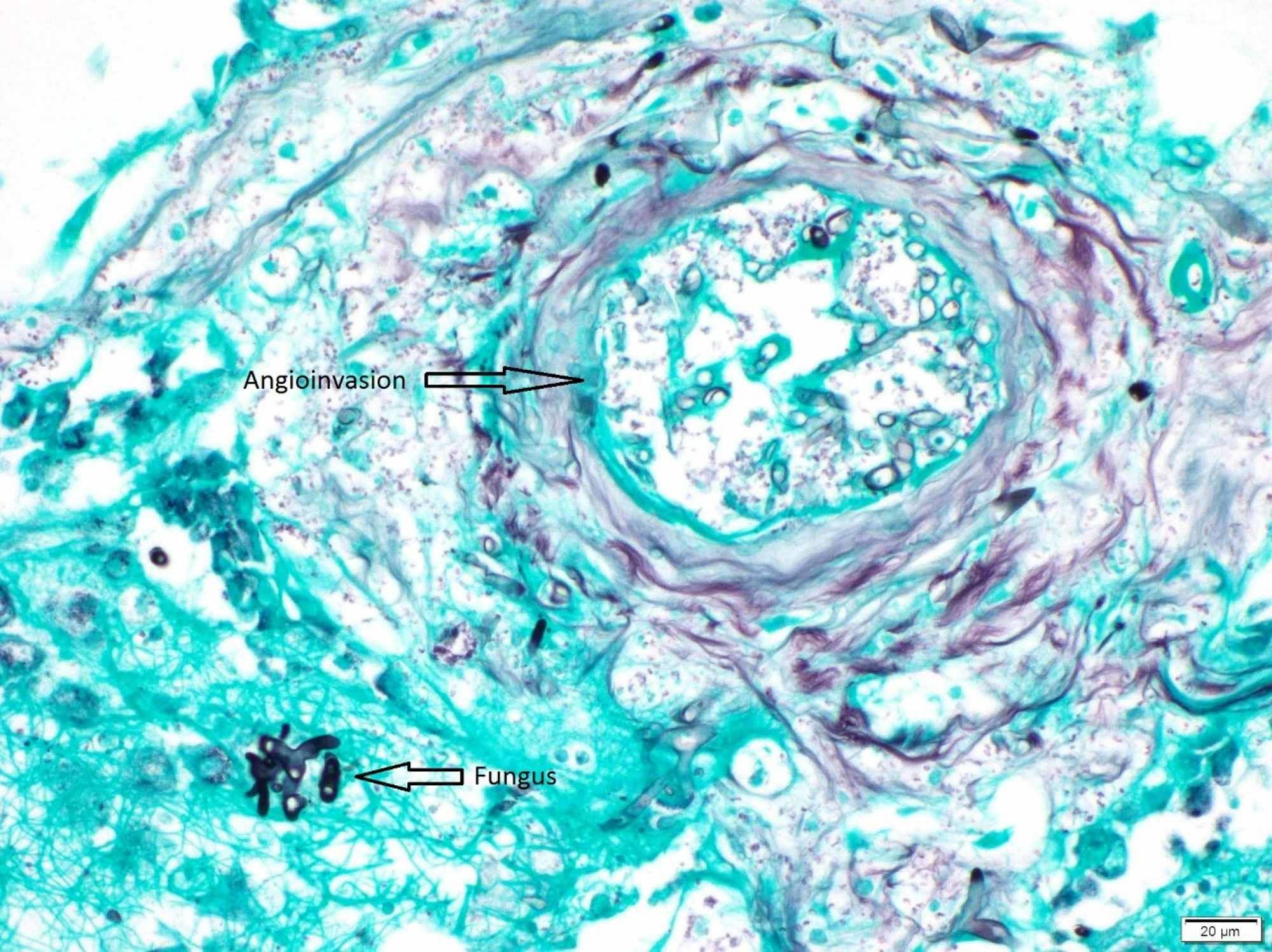
Left lung nodule Gomori's Methenamine Silver (GMS) stain Angioinvasion by broad, aseptate, ribbon-like hollow hyphae with right angle branching, compatible with zygomycete species

A CT-guided biopsy from the lung nodules was performed (Figure 2), which demonstrated ribbon-like hyphae compatible with mucormycosis with pathognomonic angioinvasion finding (Figures 3-5).

**Figure 6:**
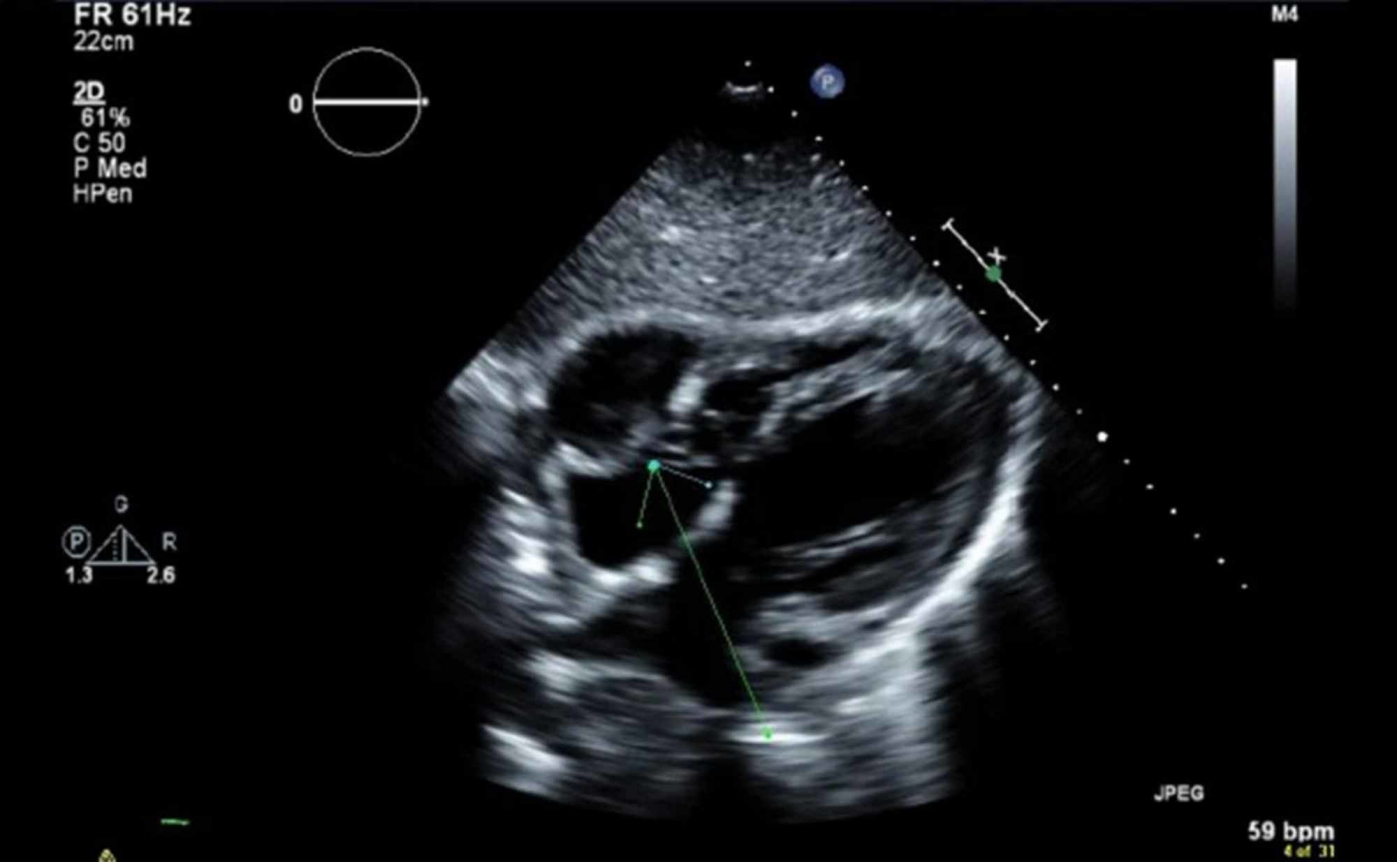
Echocardiogram Mass (arrow) inside the right atrium

CT of the face and sinus, as well as nasolaryngoscopy, showed no evidence of invasive fungal sinusitis. Echocardiography demonstrated a left ventricular ejection fraction of 45%-50% and an echogenic mass adjacent to the right atrium and right ventricle with a free mobile component (Figure 6).

**Figure 7:**
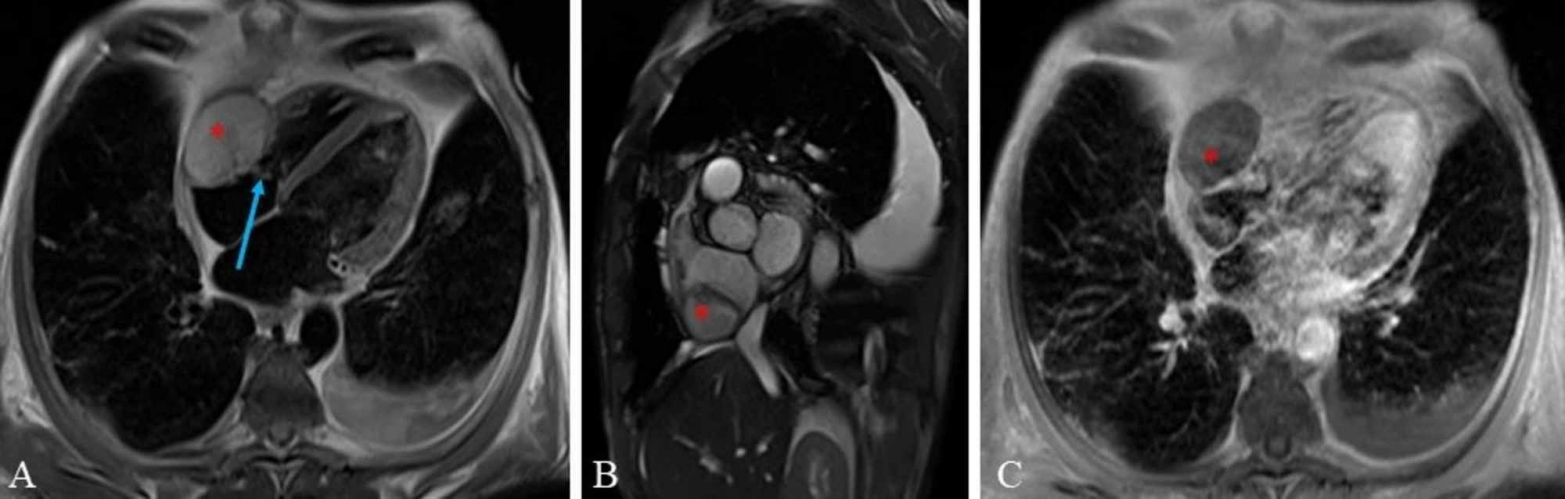
Cardiac magnetic resonance imaging (A) Four chamber view T2 dark blood magnetic resonance imaging (MRI); (B) short axis view cine steady-state free precession MRI; and (C) four-chamber view. T1 fat saturated post-gadolinium contrast MRI demonstrates a non-enhancing mass (asterisk) extending between the right atrium and right ventricle through the tricuspid valve. The mass has a friable component in the right atrium that is freely mobile (arrow). This mass was thought to represent a thrombus, with or without superinfection.

Cardiac magnetic resonance imaging showed a 5.6 x 3.8 cm intracardiac mass extending between the right atrium and right ventricle and a free mobile component in the right atrium (Figure 7).

**Figure 8:**
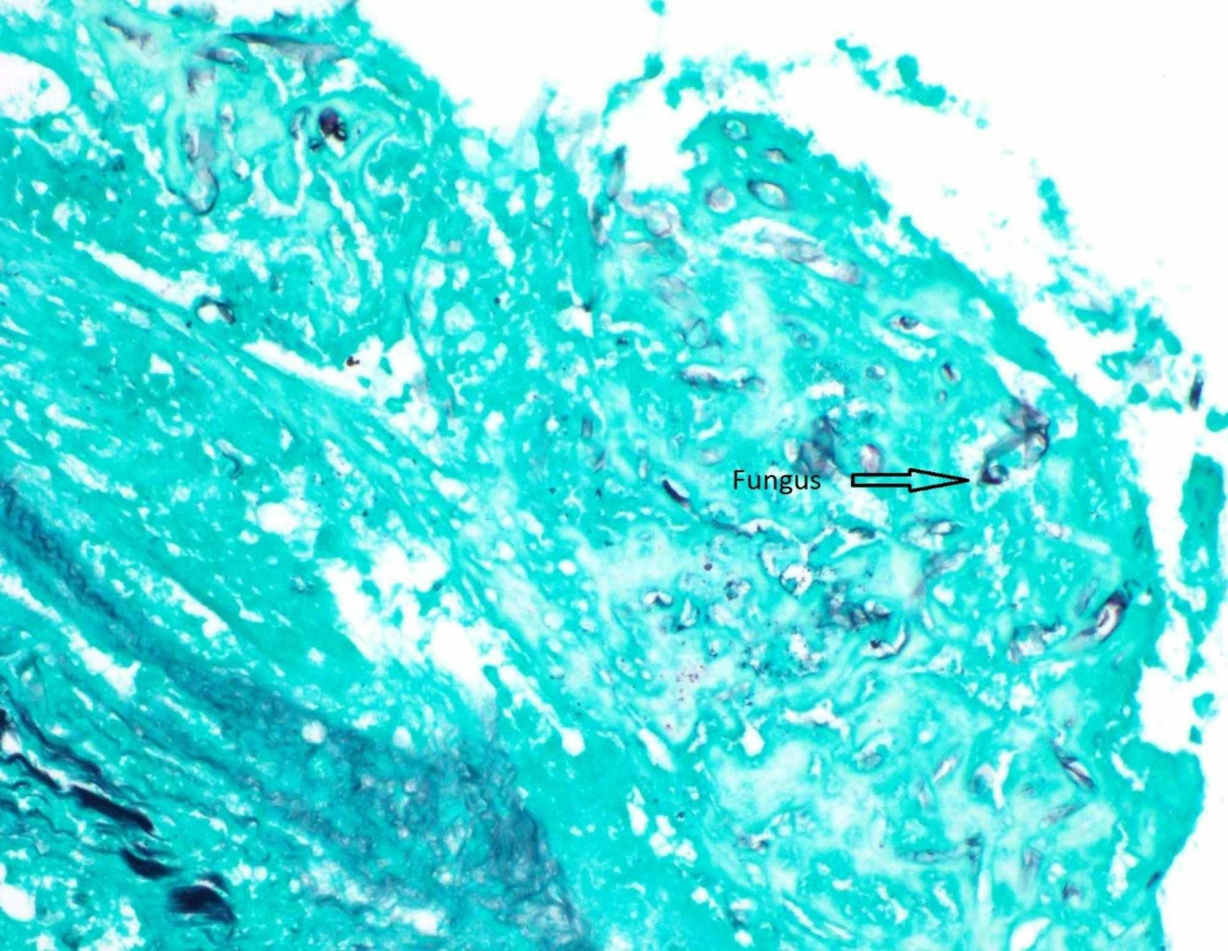
Right atrial mass Gomori's Methenamine Silver (GMS) stain 40x Degenerating fungal hyphal forms

The patient was not a candidate for surgical debridement. An echo-guided cardiac biopsy was performed to delineate the extent of disease. The biopsy showed necrotic muscle and degenerating fungal hyphae resembling the finding of pulmonary CT-guided biopsy (Figure 8).

The patient was started on liposomal amphotericin B. A repeat chest CT after two weeks of therapy showed a slight interval decrease in size in several pulmonary nodules. He was then transitioned to oral posaconazole to be continued as an outpatient till the resolution of neutropenia and surgical debridement of the cardiac mass, if ever possible, and to monitor his clinical and radiological response as per infectious disease service recommendations. He was discharged to an acute physical rehabilitation facility on 3 liters oxygen via a nasal cannula. Four weeks interval imaging showed a slow decline in the size of his pulmonary nodules and cardiac mass. Clinically, the patient did not appreciate a significant improvement in his oxygen requirement.

## Discussion

Mucormycosis is a rare opportunistic organism that can lead to invasive fungal disease. It is seen most often in immunocompromised hosts, such as those with hematological malignancies, hemopoietic stem cell transplantation, human immunodeficiency virus (HIV), and chronic steroid use. Other major risk factors include diabetes, particularly in those with ketoacidosis [7]. Individuals with hematological malignancies are more susceptible to a rapidly fatal course of disseminated disease. Despite progress in diagnosis and management, mortality rates are still as high as 70% [8]. Multiple factors can play a role in determining the morbidity and mortality of mucormycosis. In malignancy, the mortality rate has been reported as high as 66%. Once disseminated, mortality can increase to 96% [9]. For those that are treated with antifungal therapy in contrast to those who are not, survival is 70% and 3%, respectively [9]. The high mortality rate of mucormycosis can be attributed to the difficulty in diagnostic evaluation. Symptoms and radiographic findings of mucormycosis are nonspecific and microbial culture and fungal serologies are often non-diagnostic. Moreover, the availability of biomarkers that aid in the noninvasive diagnosis of mucormycosis is limited. Hence, a histopathological diagnosis obtained by invasive tissue biopsy is required for definitive diagnosis in the majority of the cases [10].

Neutrophils provide the primary defense against hyphae. Any disorder affecting their count or pathogenic and chemotactic capacity would make the host more susceptible to a pathogenic form of mucormycosis. For that reason, the timing of neutrophilic count recovery in neutropenic patients is of exceeding clinical importance [8]. The three most common clinical presentations of mucormycosis are sinusitis (39%), pulmonary (24%), and cutaneous (19%). Dissemination has been shown to develop in 23% of these cases [11]. The most common presenting symptom is fever (51%) [12]. Fever that is refractory to antibiotics and other antifungal agents can sometimes be the only presenting symptom. Hemoptysis and pleuritic chest pain are late and uncommon symptoms. In a literature review of 23 cases of pulmonary or pulmonary-plus disease, most imaging of patients had infiltrates (26%), with bilateral distribution (30.5%). Radiologic evidence of cavitation or nodules (17.39% each) and consolidation (13%) was less commonly seen [13].

Disseminated mucormycosis is a rare condition that involves two or more non-continuous organ systems. Independent risk predictors for the disseminated clinical syndrome are patients with hematological malignancies or organ transplantation or in patients on deferoxamine therapy [14]. The successful management of mucormycosis entails the early diagnosis and initiation of antifungal therapy along with surgical debridement of necrotic and devitalized tissue. Of equal importance, when possible, is the reversal and control of the underlying predisposing factors (immunosuppression, hyperglycemia, etc.) [15].

Three systemic antifungals have Mucorales activity: amphotericin and its formulations, posaconazole, and isavuconazole [16]. Amphotericin B or its lipid formulation (liposomal amphotericin), which has a better therapeutic index, remains the most reliable single agent for mucormycosis [17]. The optimal dosage and duration of therapy with amphotericin B and its lipid formulation against mucormycosis has not yet been determined. In a French prospective study comparing the efficacy and safety between the administration of a standard dose of 5 mg/kg/day and those who received a higher dose of 10 mg/kg/day, results showed no improvements in mortality or response rates observed over 12 weeks of treatment. On the other hand, renal toxicity, as shown by an elevation of serum creatinine level, was observed in 40% of patients receiving higher dosages [18]. In nonhuman neutropenic murine models, Lewis et al. proposed that the effectiveness of liposomal amphotericin B in treating pulmonary mucormycosis is dose-dependent when he compared a more effective dose of 10 mg/kg/day to a lower and less effective dose of 5 or 1 mg/kg/day [19]. In murine models, a combination of polyenes and an echinocandin was shown to have a synergistic effect on mucormycosis [20]. Retrospective studies conducted in murine models on rhinocerebral mucormycosis showed better outcomes when that combination was used [20]. Among newer triazole antifungals, only posaconazole and isavuconazole have shown efficacy against mucormycosis. Both could serve as a step-down therapy in patients who show a clinical and radiologic response or as salvage therapy in patients who are intolerant or refractory to first-line therapy with liposomal amphotericin B [18]. Only isavuconazole, a second-generation triazole, has been proposed to be used as first-line against mucormycosis with efficacy similar to amphotericin B [16].

## Conclusions

We present a case of native heart involvement as a part of disseminated mucormycosis syndrome with primary lung foci that is uncommon and often diagnosed on autopsy, antemortem diagnosis is very rare and to our knowledge, only four such cases have been reported in the literature till now. Disseminated mucormycosis syndrome is a life-threatening condition that presents both a diagnostic and a treatment challenge. Consequently, a high index of suspicion and an aggressive diagnostic and therapeutic approach with the timely start of preemptive antifungal therapy are vital factors to improve outcomes, especially in severely ill neutropenic patients with a possible infection that is refractory to broad-spectrum antibiotics.
